# Proximal Optical Sensors for Nitrogen Management of Vegetable Crops: A Review

**DOI:** 10.3390/s18072083

**Published:** 2018-06-28

**Authors:** Francisco M. Padilla, Marisa Gallardo, M. Teresa Peña-Fleitas, Romina de Souza, Rodney B. Thompson

**Affiliations:** 1 Department of Agronomy, University of Almeria, Carretera de Sacramento s/n, 04120 La Cañada de San Urbano, Almería, Spain; mgallard@ual.es (M.G.); mtpena.fl@ual.es (M.T.P.-F.); rominadesouzai@gmail.com (R.d.S.); rodney@ual.es (R.B.T.); 2 CIAIMBITAL Research Centre for Mediterranean Intensive Agrosystems and Agrifood Biotechnology, University of Almeria, 04120 La Cañada de San Urbano, Almería, Spain

**Keywords:** chlorophyll meters, flavonols, fluorescence, precision agriculture, reflectance, vegetation indices

## Abstract

Optimal nitrogen (N) management is essential for profitable vegetable crop production and to minimize N losses to the environment that are a consequence of an excessive N supply. Proximal optical sensors placed in contact with or close to the crop can provide a rapid assessment of a crop N status. Three types of proximal optical sensors (chlorophyll meters, canopy reflectance sensors, and fluorescence-based flavonols meters) for monitoring the crop N status of vegetable crops are reviewed, addressing practical caveats and sampling considerations and evaluating the practical use of these sensors for crop N management. Research over recent decades has shown strong relationships between optical sensor measurements, and different measures of crop N status and of yield of vegetable species. However, the availability of both: (a) Sufficiency values to assess crop N status and (b) algorithms to translate sensor measurements into N fertilizer recommendations are limited for vegetable crops. Optical sensors have potential for N management of vegetable crops. However, research should go beyond merely diagnosing crop N status. Research should now focus on the determination of practical fertilization recommendations. It is envisaged that the increasing environmental and societal pressure on sustainable crop N management will stimulate progress in this area.

## 1. Introduction

Nitrogen (N) fertilizer is applied in large amounts in modern vegetable production to ensure high yields [1]. Generally, only a minor part of the N applied is recovered by crops, and the excess N is susceptible to loss to the environment where it is associated with various environmental problems. Excess applied N can be leached below the root zone or lost in run-off [2,3,4], resulting in the accumulation of nitrate (NO_3_^−^) in natural water bodies [5,6]. Increased NO_3_^−^ concentrations in water bodies are associated with human health issues [7] and eutrophication [3]. Additionally, excess applied N can be lost to the environment through nitrous oxide (N_2_O) emissions, which contribute to global warming, and through ammonia (NH_3_) volatilization, which contributes to N enrichment of natural ecosystems [8,9,10,11].

The protection of the environment has become a necessary consideration for intensive agriculture and horticulture. Nitrogen fertilizer management based on insuring production by using appreciable excessive amounts is no longer acceptable to modern societies [12,13]. The ongoing increase in environmental concern and related constraints on agriculture is reflected in legislation, such as the European Union (EU) Nitrates Directive [14] and Water Framework Directive [15] that aim to protect water quality across Europe by preventing nitrates from agricultural sources polluting ground and surface waters.

For optimal crop N management that ensures high production, while minimizing N losses to the environment, N fertilizer should be applied to match crop demand and to take into account N supplied by other sources, such as soil mineral N present at planting, and N mineralized from soil organic N, manure, and crop residues [9,13,16,17,18]. Optimal N fertilization of vegetable crops, in respect to both quantity and timing, would benefit appreciably from an accurate assessment of crop N status i.e., whether the crop N supply has been deficient, adequate, or excessive, and the degree of deficiency or excess. Traditional approaches to vegetable crop N management have been fertilizer recommendation schemes based on soil analysis, such as the Nmin or KNS (crop accompanying Nmin system) methods or estimation of the soil N supply as in the RB209 schemes in the United Kingdom [13,19,20]. Additionally, tissue analysis i.e., total N analysis of leaves, has been used, but mostly for diagnosis of problems [19,20]. Although these procedures are useful, in particular the soil analysis based recommendation schemes for estimating total requirements, the analyses are time-consuming and do not yield results quickly in the field so as to make fertilizer recommendations on time [21]. This is particularly so for vegetable crops that receive frequent N application through fertigation, which is being increasingly used with intensive vegetable production throughout the world [18,22,23]. With fertigated crops, ideal N management would involve rapid and frequent assessment of crop N status, enabling rapid adjustment of the N being applied [24].

A promising approach for the rapid and periodic assessment of crop N is the use of proximal optical sensors [25]. They could also be used for frequent assessment where fertigation is used [13,24,26]. Proximal optical sensors are a form of remote sensing in which the sensors are positioned either in contact with or close to the crop [13,25]. Optical sensors do not directly measure the N content in plant tissue, but provide either: (a) Indices of radiation measurement or (b) indirect measurements of indicator compounds that are sensitive to the crop N status [19,27,28]. These sensors determine crop N status indirectly and non-destructively [27,28,29]. The major advantages of optical sensors are that they can be used any time during the growth cycle on commercial farms, they require limited labor, and they can be integrated into fertilizer decision making procedures [21,30,31]. Some sensors are limited to individual spot measurements, while others have continuous on-the-go capabilities that enable large representative surface areas of foliage to be measured. These characteristics make them well-suited to practical assessment of crop N status [9,21,32].

This manuscript reviews the use of different proximal optical sensors for assessing crop N status and for N management of vegetable crops. To date, most of the evaluations of proximal optical sensors for crop N management have been with cereals for single side-dress N fertilizer applications at a given crop development stage or age. There have been a number of publications on the use and applicability of optical sensors for N management of vegetable crops; however, this information is dispersed, involving different crops and different sensors, and has never been thoroughly reviewed. Vegetable production has an inherently high nutrient pollution potential because of the intensive management and high value of the produce [13]. The high value of the produce encourages risk adverse management practices, such as high fertilizer rates and frequent irrigation [8,33,34]. Additionally, many vegetable crops are shallow rooted, which limits N uptake at depth, increasing the possibility of NO_3_^−^ leaching [2,35,36].

The three types of N-sensitive proximal optical sensors, suitable for crop N management applications, that have been most used with vegetable crops are reviewed here: Chlorophyll meters, reflectance sensors, and fluorescence-based flavonols meters (Table 1). For each type of sensor, the scientific basis, principle of operation, and measurement considerations are described and the main research conducted with vegetable crops is reviewed. Notable characteristics and/or results with individual sensors are also described. The final section deals with the practical application of proximal optical sensors for N management of vegetable crops.

## 2. Chlorophyll Meters

The first group of optical sensors studied for crop N management was chlorophyll meters, which indirectly estimate the relative chlorophyll content per unit of a leaf surface area [37]. Chlorophyll is an N-sensitive compound that is strongly related to leaf N content [27,38,39] because most leaf N is contained in the photosynthetic apparatus and enzymes involved in photosynthesis [40]. Most chlorophyll meters are hand-held devices that clip onto or are placed close to the leaf surface. Chlorophyll meters measure a dimensionless value that is strongly related to the actual amount of chlorophyll [37,41,42].

**Figure 1:**
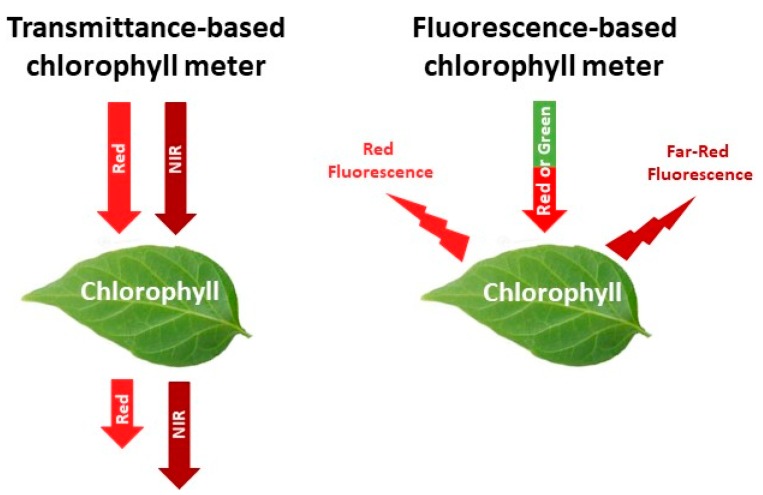
Functioning of two different types of chlorophyll meters used to estimate leaf chlorophyll content.

Most chlorophyll meters estimate the relative leaf chlorophyll content by measuring the absorbance and transmittance of radiation by the leaf of: (1) Red radiation, which chlorophyll absorbs; and (2) near infra-red (NIR) radiation, which chlorophyll transmits [19,43] (Figure 1). Absorbance of red radiation increases with chlorophyll content, resulting in higher chlorophyll meter values [39,44,45]. These sensors are referred to as transmittance-based chlorophyll meters. There are currently several commercially available transmittance-based chlorophyll meters, including the SPAD-502 and N-tester, which are almost identical, the more recent and low-cost atLEAF+ sensor, or the MC-100 Chlorophyll Concentration Meter (Table 1).

A different approach to estimating the relative leaf chlorophyll content is through the chlorophyll fluorescence emission ratio of red and far red radiation [28,46,47,48] (Figure 1). The ratio of red to far-red chlorophyll fluorescence depends largely on the chlorophyll content; due to re-absorption of red chlorophyll fluorescence within the leaf, this ratio decreases with increasing chlorophyll content [46]. The sensors that use this approach are referred to as fluorescence-based chlorophyll meters [28]. One example with potential for use in crop N management is the MULTIPLEX sensor (Table 1).

Several chlorophyll meters are currently commercially available (Table 1) and they differ from one another with respect to one or more of the following: The measuring principle (i.e., transmittance versus fluorescence), the wavelengths used, the measurement units, and the calibration equations used to convert electrical signals into measurement units. This diversity of approaches complicates comparisons between measurements made with different chlorophyll meters [42,49,50].

Numerous studies have reported strong relationships between chlorophyll meter measurements and the extractable chlorophyll concentration in the form of curvilinear relationships with a degree of saturation of measurements at high chlorophyll contents [41,51,52]. Standard chlorophyll content versus chlorophyll meter relationships for several different species have been reported [42,50]. However, different relationships for different species [40], and for different cultivars within the same species [19], have also been suggested.

### 2.1. Measurement with Chlorophyll Meters—Practical Issues

All chlorophyll meters are, generally, easy to use, do not require any particular training of users, and make measurements very rapidly with no or very little data processing [53]. Calibration is a simple procedure conducted before measuring. Operationally, chlorophyll meters are very well-suited for on-farm use to provide rapid assessment of vegetable crop N status. Protocols for the use of chlorophyll meters to aid crop N management have been developed [21,27,54,55].

Given the relatively small sampling area (from 6 mm^2^ of SPAD up to 1250 mm^2^ of MULTIPLEX), appreciable repetition and strict sampling protocols are required for representative measurement. Measured values should be the average of, at least, several individual readings per field (or from replicated experimental plots). Normally, each individual measurement is made on a unique plant [53]. It is recommended that measurements be made on the most recent fully expanded and well-lit leaf, between the stalk (stem) and leaf tip, midway between the margin and mid-rib, on the adaxial (top) side of the leaf, and on plants randomly selected from the center of fields/plots. Care should be taken to avoid damaged leaves and leaves with moisture [56].

The diverse leaf anatomy of vegetable plants can present challenges for measurement with chlorophyll meters. Westerveld et al. [57] reported that SPAD measurement was affected by the leaf morphology of cabbage, onion, and carrot leaves. For example, leaf veins should be avoided [57]. Light transmittance through the interior of tube-shaped onion leaves affected the area being measured by the SPAD meter, making it necessary to protect measured areas from direct sunlight [57]. Additionally, pressure from the leaf clip system can cause extrusion of sap from onion leaves, requiring more frequent cleaning [57]. Finely-dissected carrot leaves were difficult to measure because the individual leaflets had a similar size to the small measurement area of the meter used [57]. For composite leaves, such as tomato, it is necessary to clearly define the leaflet and the position on the leaflet to be sampled [55]. On large leaves, such as of cucurbits, it is necessary to clearly define the measurement position [58,59]. In all cases, clearly-defined and easily identifiable positions on leaves are required to standardize measurement.

Irradiance conditions can influence chlorophyll meter measurements [60]. Chlorophyll meter values in wheat were lowest at high irradiance (midday), and highest at low irradiance at dusk and dawn [61]. Reducing photosynthetically active radiation (PAR) from 1100 to 600–650 µmol m^−2^ s^−1^ increased chlorophyll meter values in maize [62]. To enable comparison of sequences of measurements, the time of day and the irradiance conditions should be standardized. The general recommendation is for chlorophyll meter measurements to be made at the same time of day at noon solar time under clear skies [53].

Agronomic factors affecting crop vigor and appearance [12,53,60,63], such as water stress, nutrient deficiencies, and cultivar [64], may influence chlorophyll meter measurements. Such possible effects should be ruled out when interpreting chlorophyll meter data. In greenhouse-grown vegetable production, favorable crop growing conditions are, generally, maintained regarding irrigation, nutrition, climatic conditions, and pest control, which will reduce the possible effect of these factors.

### 2.2. Evaluation of Crop N Status Using Chlorophyll Meters—General Experience

Since the development of chlorophyll meters for monitoring rice N status in the early 1980s [19,37], considerable research has evaluated their use with various crop species, mostly with cereals, such as maize [39] and wheat [63]. Most research has been conducted with transmittance-based chlorophyll meters [19]. Recently, there has been increasing interest in fluorescence-based chlorophyll meters [65,66].

In a review article, Fox and Walthall [19] concluded that chlorophyll meter measurements were, generally, strongly related to leaf and crop N content, with better relationships being obtained when used for single cultivars than across cultivars within a species. Many studies have reported linear relationships between chlorophyll meter values versus crop/leaf N content for measurements made at a particular time or growth stage [38,50,55,67,68]. However, some studies have reported a plateau response, where, at relatively high N contents, the linear relationship tends to “flatten out” [32,53]. This plateau response is similar to that reported at relatively high leaf chlorophyll contents, which was referred to in Section 2.1.

There is consistent evidence that chlorophyll meters can become partially saturated at high N and chlorophyll contents [37,41,42,69]. However, often the partial saturation response does not occur, and linear relationships are observed between chlorophyll meter readings and leaf chlorophyll or leaf/plant N content [28,38,70,71]. It is not clear exactly what conditions (e.g., species, chlorophyll, and N contents) are causing partial saturation to occur and also what the conditions are when saturation does not occur [13].

### 2.3. Evaluation of Crop N Status Using Chlorophyll Meters in Vegetable Crops

Chlorophyll meters have been successfully used to assess crop N status of vegetable crops in numerous studies with species, such as fresh tomato [55,72], processing tomato [68,73], muskmelon [58], cucumber [59], sweet pepper [42], or potato [53,54].

Numerous field studies were carried out with the potato in Italy, Belgium, United Kingdom, and The Netherlands to evaluate several chlorophyll meters (SPAD-502 and Hydro N-Tester) for monitoring crop N status, as part of the EU action Efficiency in Use of Resources for Optimizing Potato Production (EUROPP) [53]. In these studies, linear relationships were consistently obtained between chlorophyll meter values and the leaf and total crop N contents; these relationships for the potato were further confirmed by Majic et al. [74]. Farneselli et al. [73] reported strong relationships between SPAD values and crop N content in processing tomatoes. These results were further supported by Güler and Büyük [75]. Very similar results were found in tomatoes for fresh consumption [55]. These authors reported strong relationships between SPAD measurements, crop N content, and the Nitrogen Nutrition Index (NNI) throughout most of the tomato crop cycle. The NNI is an effective and established indicator of crop N status [12], calculated as the ratio between actual crop N content and the critical crop N content, i.e., the minimum N content necessary to achieve maximum growth of a crop [76]. Similarly, strong relationships between SPAD measurements and crop and leaf N content were found in individual phenological phases of the tomato [72,77]. Contrasting results to those mentioned both for processing and fresh-consumption tomatoes were found by Ulissi et al. [78] in the processing tomato, where weak relationships between SPAD values and plant N content were likely caused by the narrow range of plant N contents evaluated.

**Figure 2:**
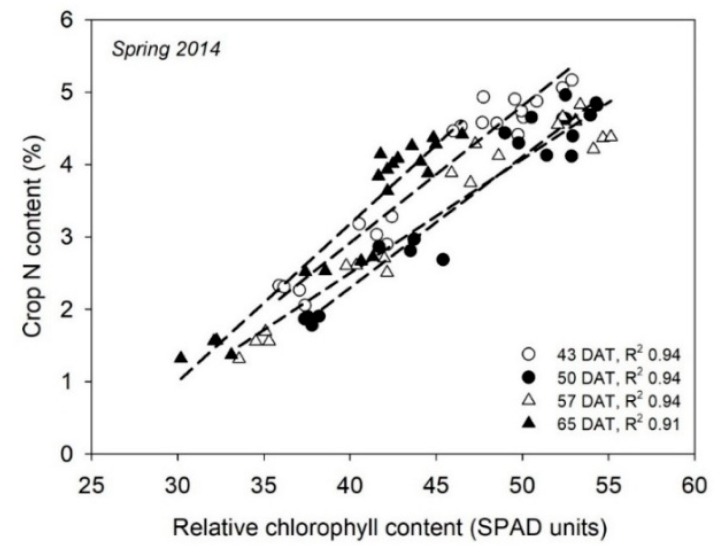
Relationships of SPAD units to crop N content from a greenhouse-grown cucumber crop carried out in southeast Spain. DAT is days after transplanting and R^2^ is the coefficient of determination.

A narrow range of crop N content may also have been responsible for the weak relationships between SPAD measurements and plant N content reported for greenhouse-grown cucumbers [75]. This latter work contrasted with the strong relationships between SPAD measurements and crop N content (Figure 2), between SPAD and NNI, and between SPAD measurements and yield in two greenhouse-grown cucumber crops [59]. In addition, there were strong relationships within most phenological phases, regardless of the growing season of the cucumber crop [59]. In another greenhouse-grown cucurbit crop, muskmelon, chlorophyll measurements of the SPAD-502 were strongly related to crop N content and NNI throughout most of the crop cycle [58].

## 3. Reflectance Sensors

**Figure 3:**
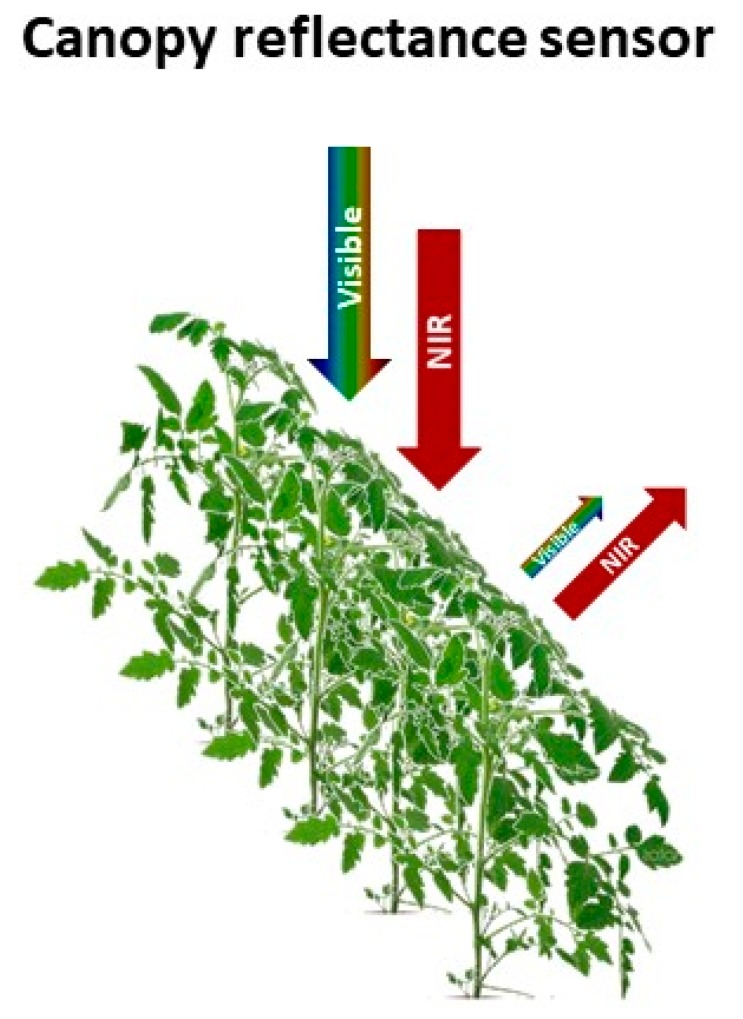
Schematic representation of the functioning of active canopy reflectance sensors. Differential reflectance of visible and near-infra red radiation (NIR) is used to calculate vegetation indices.

During the last 20 years, there has been substantial research on the use of proximal reflectance sensors to assist with crop N management [9,19,27,79]. Reflectance sensors provide information on the crop N status by measuring specific wavelengths of radiation absorbed and reflected from crop foliage [80,81,82,83]. In proximal canopy reflectance, sensors are positioned relatively close to the crop (e.g., 0.4–3.0 m from the crop canopy). Plant tissue normally absorbs approximately 90% of the visible radiation (390 to 750 nm) and reflects approximately 50% of the NIR (750 to 1300 nm) [81]. The degree of absorbance and reflectance in the visible and NIR portions of the spectrum varies with crop N content, thus, providing information on the crop N status (Figure 3); N-deficient crops, generally, reflect more visible and reflect less NIR than N-sufficient crops [39,82].

The wavelengths selected for N assessment are chosen because of their sensitivity to changes of chlorophyll status, foliage density, and biomass that accompany N deficiency. These often correspond to four narrow bands, in order of importance, centered around 675 nm (red absorption maxima), 905 nm (NIR reflection peak), 720 nm (mid portion of the red-edge), and 550 nm (green reflectance maxima) [21,78,84,85]. To increase the sensitivity to specific biophysical characteristics and reduce variability, spectral vegetation indices that combine spectral reflectance from 2–3 wavelengths are calculated [86,87] (Table 2). The Normalized Difference Vegetation Index (NDVI) [88] is probably the most widely-used. While the simple ratio indices and many normalized indices must be measured directly on the crop canopy, there are some specific indices that are able to distinguish vegetation from soil (e.g., the Soil Adjusted Vegetation Index (SAVI) [89]). Details of over 40 spectral vegetation indices can be found in Bannari et al. [86] and Ollinger [83]. Some of the most commonly-used vegetation indices are in Table 2.

Reflectance sensors are classified as passive or active sensors depending on whether they have their own light source. Passive sensors normally have two sets of photodetectors, one set measures incident radiation above the crop canopy, and the other measures radiation reflected from the canopy; the measurement of incident radiation is used by the sensor to consider different irradiance conditions while operating. Newer, active sensors have a light source that emits both visible radiation and NIR. By modulating the light source, active sensors can distinguish reflected radiation from their own light source from that derived from ambient radiation [100], and so active sensors can be used in any irradiance conditions [101]. Examples of active sensors are the various Crop Circle and GreenSeeker sensors, and the N-Sensor ALS (Table 1). There are various models of the Crop Circle and GreenSeeker sensors, with both having simpler, cheaper, and hand-held models that are well-suited to manual use with vegetable crops [13]. The more expensive models can, generally, be used for continuous data recording, for which they are often mounted on tractors and connected to GPS systems for field mapping. They are mostly used in this configuration for automatic variable rate application of mineral fertilizer.

### 3.1. Practical Issues with Proximal Canopy Reflectance Sensors

Crop reflectance measurements are one of the most promising methods for monitoring the N status of vegetable crops. One of their main advantages is that in an individual measurement they integrate the measurement of a much larger area than that measured by leaf level chlorophyll meters [32,55]. Many reflectance sensors can make continuous measurements on-the-go at walking speed, if hand-carried, or at tractor speed, if fitted onto a tractor. Some of the more sophisticated modern sensors only permit continuous measurement (e.g., Crop Circle ACS, GreenSeeker); some of the more recent sensors are capable of either individual or continuous measurement (e.g., GreenSeeker Handheld, RapidScan CS-45 (Table 1)). Continuous measurement is made by making several or more passes of at least several meters along crop rows, with the sensor typically positioned horizontally above the crop canopy and facing downward or with the sensor positioned vertically and parallel to the crop row, with the sensor facing the upper part of the crop foliage, at a minimum distance to the canopy [55,102]. In experimental studies, appreciable areas of the canopy can be measured in each replicate plot. To optimize sensor performance, consistency in the sensor angle in relation to the measured surface [103] and in the sensor height/distance to the target canopy/foliage [101,104] are required. These issues are considered to be important contributing factors to measurements made with different sensors, which often have different sampling size areas and positioning requirements, and differ when compared on the same crop [105]. These factors should be considered when comparing reflectance measurements obtained from different sensors.

A favorable practical characteristic of active proximal reflectance sensors is that no special requirement for uniform irradiance is required, which enables measurement under all ambient irradiance conditions [100,101,106]. However, for passive reflectance sensors, uniform irradiance conditions are highly recommended; in these cases, strategies to compensate for any drift in reflectance measurements during the day may be needed when using sensors to control the application of mineral N fertilizer [107].

In addition to crop N status, reflectance measurements and vegetation indices may be influenced by abiotic and biotic conditions, such as water status, irradiance and diseases, and pests [27,39,108,109]. For monitoring crop N status, it is necessary to exclude possible influencing factors that may confound reflectance measurements [19,110,111]. However, in greenhouse vegetable production, where crops are grown under optimal conditions (irrigation, nutrition, climatic conditions, and pest control), such factors are likely to be of minor importance.

### 3.2. Evaluation of Crop N Status Using Reflectance Sensors—General Experience

Reflectance-based sensing of crop N status has shown promising results in many studies [19,80,87]. Most research has been conducted with wheat [112,113,114] and maize [79,100,115,116]. NDVI has been one of the most used vegetation indices for crop N management [19,80]. There has been an appreciable adoption of proximal canopy reflectance sensors for variable rate N fertilizer application with cereal crops [117,118,119,120,121,122].

### 3.3. Evaluation of Crop N Status Using Reflectance Sensors in Vegetable Crops

Several studies have examined proximal canopy reflectance sensors in tomato crops, both for determinate processing tomatoes and for indeterminate tomatoes for fresh consumption. Gianquinto et al. [123] compared several vegetation indices, derived from measurements made with the MSR-87 Multispectral Radiometer, to assess the N status and yield of open field processing tomatoes, and observed that GVI and GNDVI were the most effective vegetation indices. Working with greenhouse-grown indeterminate tomatoes and the Crop Circle ACS 470 sensor, Padilla et al. [55] reported that the NDVI and RVI indices were the most effective vegetation indices to assess crop N status, and that these two indices were slightly more sensitive than the GNDVI and GVI. The better performance of NDVI and RVI in indeterminate tomatoes contrasted with the better performance of GNDVI and GVI in processing tomatoes [123]. Because of the different approaches to assess crop N status, the studies of Gianquinto et al. [123] and Padilla et al. [55] with tomatoes are not directly comparable; the former author considered leaf N content, while the latter author considered whole plant N content. Additionally, different canopy reflectance sensors were used. However, taken together, these two studies suggest that NDVI, RVI, GNDVI, and GVI are sensitive indices of the crop N status of tomatoes.

**Figure 4:**
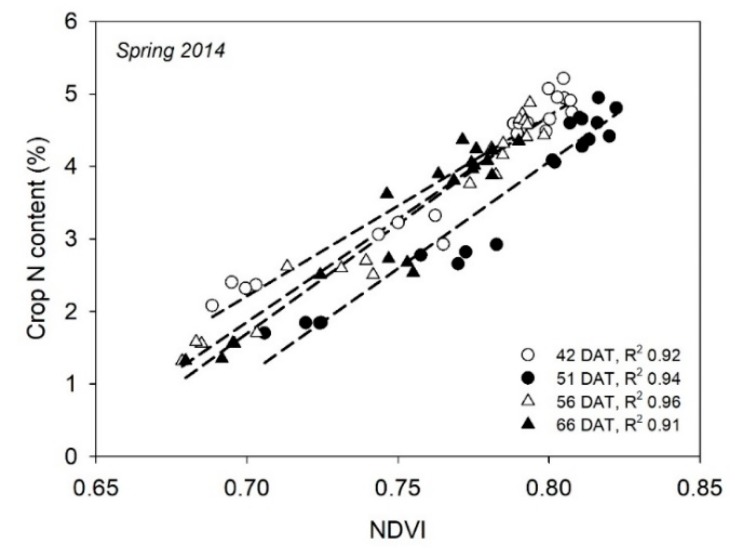
Linear relationships of the Normalized Difference Vegetation Index (NDVI), measured in the upper part of the crop canopy with a Crop Circle ACS 470 sensor, to standing crop N content from a greenhouse-grown cucumber crop in Spring 2014 carried out in southeast Spain. DAT is days after transplanting and R^2^ is the coefficient of the determination of linear regression.

In greenhouse, hydroponic-grown cucumbers, using a custom-made CCD camera, NDVI, GNDVI, and RVI were strongly related to the leaf N status of upper leaves throughout the crop cycle [124]; the strongest relationships were with NDVI. In greenhouse, soil-grown cucumbers, Padilla et al. (unpublished) reported that the NDVI, measured with the Crop Circle ACS 470 sensor, was strongly related to the crop N content (Figure 4). Also in soil-grown cucumbers and with the same sensor, Padilla et al. [125] showed that nine vegetation indices, including NDVI, GNDVI, RVI, and GVI, were strongly related to the crop NNI and yield. These relationships were observed throughout both an autumn cycle and a spring cycle crop, and when using combined data sets of the two crops. Most of the nine vegetation indices examined were good estimators of the crop N status and yield in cucumbers; the most effective indicators were GNDVI and GVI [125]. With another cucurbit crop, muskmelon, also grown in soil in a greenhouse, Padilla et al. [58] reported strong linear relationships between several vegetation indices, measured with the Crop Circle ACS 470 sensor, and the crop N status. The NDVI most accurately estimated the crop N status in the central stages of the crop; generally, similar results were obtained with GNDVI, RVI, and GVI.

The ability of the canopy reflectance to assess broccoli N status was evaluated by El-Shikha et al. [109] using Intor optical sensors (Intor Inc., Socorro, NM, USA). These authors reported that CCCI was highly sensitive to the crop N status, and that RVI, NDVI, and GNDVI were similarly sensitive.

## 4. Fluorescence-Based Flavonols Meters

**Figure 5:**
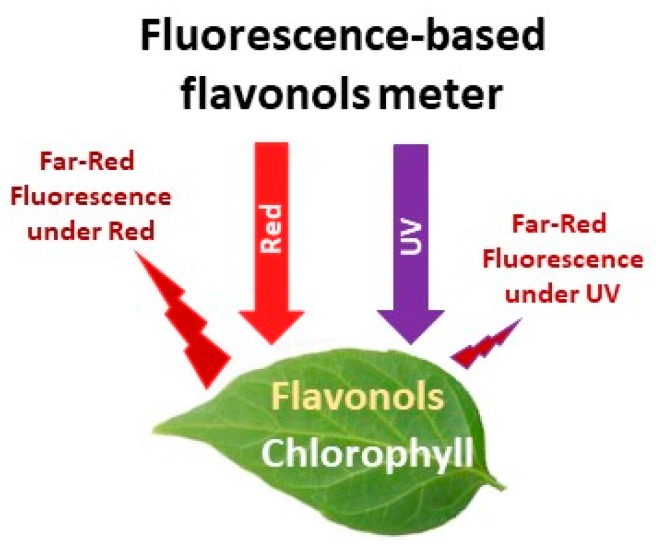
Schematic representation of the functioning of fluorescence-based flavonols meters by using the chlorophyll fluorescence screening method.

A newer approach that has been used to monitor the crop N status proximally is based on the estimation of relative flavonols content from fluorescence measurements [28]. Flavonols are a class of polyphenolic compounds, which are carbon-based secondary metabolites whose content in leaves increases under conditions of lower N supply [126,127]. Leaf flavonols content is, generally, inversely related to leaf chlorophyll content [38]. Relative flavonols content is estimated by flavonols meters using the chlorophyll fluorescence (ChlF) screening method [128,129,130]. The measuring principle is based on the emission of fluorescence in the red to far-red region of the light spectrum by mesophyll chlorophyll when excitated with red and ultra violet (UV) radiation [46,131,132]. Flavonols that accumulate in the leaf epidermis absorb appreciable amounts of UV radiation and reduce the emission of far red chlorophyll fluorescence under UV excitation, but do not affect the emission of far red chlorophyll fluorescence under red excitation (Figure 5). The flavonols content is then estimated by comparing far red chlorophyll fluorescence under red and UV excitation [38,130,133]. Flavonols meters measure a dimensionless value that is strongly related to the actual amount of flavonols [28,43,134].

Two fluorescence-based flavonols meters are most commonly used for estimation of flavonols content, being the DUALEX and MULTIPLEX (Table 1). The DUALEX is a leaf-clip instrument that measures fluorescence on a small diameter section of the leaf, whereas the MULTIPLEX is a hand-held proximal sensor that measures at 10 cm from the leaf and a surface area of 4 to 8 cm in diameter [48,102].

### 4.1. Practical Issues with Fluorescence-Based Flavonols Meters

One of the advantages of fluorescence-based flavonols meters for crop N monitoring is that the fluorescence measurement is essentially chlorophyll-based, and the soil does not interfere with the fluorescence signal the sensor receives [28]. This is unlike canopy reflectance sensors where reflectance signals from bare soil can complicate measurements in early growth stages of the crop and for widely spaced crops [135]. A major disadvantage of fluorescence-based flavonols meter measurements is that they are made at leaf level on single or multiple leaves; therefore, appreciable replication is required to overcome the heterogeneity associated with individual leaf measurements. This also prevents integration of the measurement with canopy depth/height, which occurs with canopy reflectance. The MULTIPLEX sensor can be used on-the-go for continuous measurement along the crop canopy [48,70]. However, maintaining a fixed distance to the foliage, which is fundamental for consistent measurement [134], is practically difficult given the bulky volume and weight of the sensor. MULTIPLEX measurements may also be affected if there is not full leaf coverage of the measured area [134].

Measurements with fluorescence-based flavonols meters should be taken on a regular basis or at critical stages of the crop on representative plants randomly selected from the center of representative areas of farms or within plots in research studies. Measurements are made on the most recent fully expanded and well-lit leaf, between the stalk and the tip of the leaf, and midway between the margin and the mid-rib of the leaf. Care should be taken at collection and the measuring of damaged leaves should be avoided. Measurements should be taken from both adaxial (top) and abaxial (bottom) sides of the leaf for more accuracy [136] and where the whole flavonols content of the leaf is to be quantified [38,43]. However, measurement on only one side is a more rapid and convenient method for on-farm measurement [38]. For small, light, hand-held, leaf-clip sensors, such as the DUALEX, flavonols measurement on both sides of the leaf is straightforward and feasible for on-farm measurement. However, for heavier and larger, non-contact sensors, such as the MULTIPLEX, single-sided adaxial measurements are more convenient and less disturbing to the crop than double-sided measurement [66]. Given that the flavonols content in the adaxial and abaxial sides of the leaves are highly correlated [38,137], measurement on one side appears to provide similarly effective monitoring of the crop N status as does dual sided measurement [58,66].

The time of day does not affect flavonols content estimated with flavonols meters [28,38,48]. Flavonols meters can be used without restrictions as to the time of the day chosen for measurement. However, flavonols content of a given crop changes inter-seasonally, concurrent with changes in solar irradiance [138,139], especially during long cycles that span different seasons [66]. Flavonols’ production in plants is increased in response to other stresses, such as water stress and radiation [138,139] or salinity stress [140]. For the measurement of flavonols to be effective for assessing the crop N status, it is necessary to rule out these additional flavonols-inducing factors. As previously mentioned regarding the other types of proximal optical sensors, in intensive vegetable production, crops are generally grown under optimal conditions regarding irrigation. In most vegetable crops, salinity stress is unlikely. It is likely that for most vegetable crops water and salinity stress are unlikely to be sufficient to confound flavonols measurement. Nevertheless, care should be taken to ensure that increases in flavonols are not due to factors other than the N supply.

### 4.2. Evaluation of Crop N Status Using Fluorescence-Based Flavonols Meters—General Experience

Even though flavonols meters are a recently developed approach for the on-farm monitoring of crop N status; data are rapidly accumulating that demonstrate their potential for crop N management. Research with flavonols meters has been conducted with a wide range of crop types and species, such as maize [48,141,142,143], wheat [38,144], turfgrasses [65,145], paddy rice [137], vineyards [102], and ornamental woody plants [146]. In a recent review, Tremblay et al. [28] highlighted that flavonols content and the Nitrogen Balance Index (NBI) [38] were the two most suitable indicators for the assessment of crop N status when using flavonols meters. NBI is the ratio between chlorophyll and flavonols contents, and, because of the opposing relationships of chlorophyll and flavonols to the plant N status, their ratio was proposed as an indicator of crop N status [28,38]. Chlorophyll content is often estimated with a separate chlorophyll meter, such as SPAD [38,58], but it is also possible to use the same sensor, DUALEX or MULTIPLEX, for simultaneous estimation of chlorophyll and flavonols content [66]. For chlorophyll content estimation, DUALEX uses light transmittance and MULTIPLEX uses chlorophyll fluorescence.

### 4.3. Evaluation of Crop N Status Using Fluorescence-Based Flavonols Meters in Vegetable Crops

Most of the research with flavonols meters has been conducted with maize [48,141,142,143] and wheat [38,144], and experience with vegetable crops is more limited. Tremblay et al. [147] reported that flavonols content and NBI, measured with DUALEX in broccoli, were sensitive to N fertilization. The use of flavonols content and NBI to assess crop N status in two potato varieties was evaluated by Abdallah and Goffart [148], using both DUALEX and MULTIPLEX. In this work, NBI was the more sensitive indicator of potato N status.

**Figure 6:**
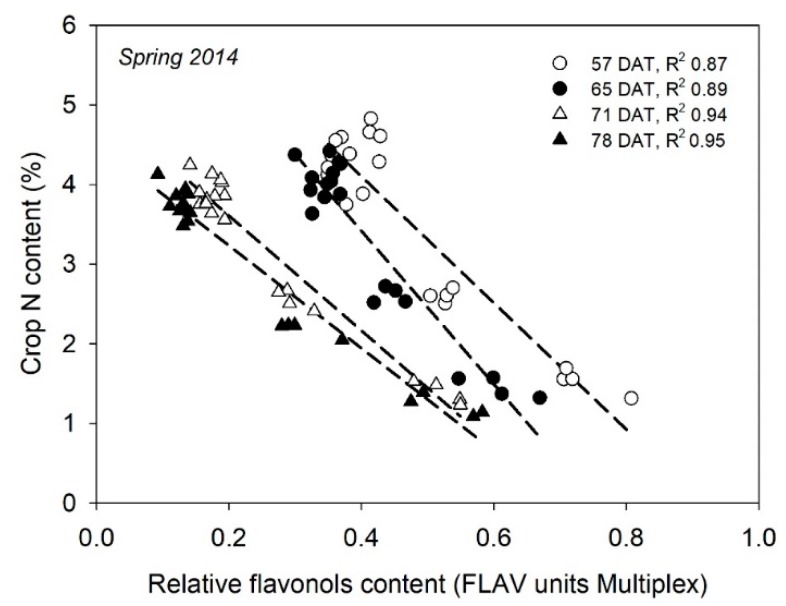
Linear relationships of leaf relative flavonols content (FLAV units of the MULTIPLEX sensor) to crop N content from a greenhouse-grown cucumber crop in Spring 2014 carried out in southeast Spain. DAT is days after transplanting and R^2^ is the coefficient of the determination of linear regression.

A number of studies have been conducted with greenhouse-grown cucurbits in soil. In muskmelon, flavonols content and NBI, measured with DUALEX, were very strongly related to crop N content and NNI for most of the crop cycle [58], suggesting that these measurements have potential to accurately estimate crop N status throughout a muskmelon crop. In cucumbers, Padilla et al. (unpublished) reported strong relationships between flavonols content, measured with MULTIPLEX, and crop N content in all dates of measurement (Figure 6). In two cucumber crops, Padilla et al. [66] found very strong relationships between both flavonols content and NBI, measured with MULTIPLEX, with crop NNI for cucumber crops during autumn and spring growing cycles. However, there was a notable difference in the flavonols content between the spring and autumn grown crops. for equivalent crop N status, that prevented the derivation of common relationships for both cycles [66].

## 5. Responses of Proximal Optical Sensors at Excessive Crop N Status

Given that excessive N application is a common occurrence in intensive vegetable crops, the ability of proximal optical sensors to detect an excessive N supply is an important practical consideration [13,66]. Under an excessive N supply, the amount of N taken up by the crop can exceed the minimum amount necessary to achieve maximum growth, resulting in a luxury N uptake [149]. There appears to be differences among vegetable species in the occurrence of luxury N uptake and, where it does occur, in the degree of luxury N uptake [13]. Appreciable luxury N uptake was observed in the muskmelon [58], cucumber [66], and tomato [55]. Species that have little luxury N uptake will not accumulate excessive N after N sufficiency has been achieved, even when increasingly excessive N is applied. In these species, it cannot be expected that optical sensors will be able to distinguish excess N in the crop.

Where luxury N uptake does occur, optical sensors may respond to an excessive N supply. Measurements from optical sensors did not exhibit a plateau response, but tended to increase at increasingly excessive N supplies and crop N status in the muskmelon, tomato, and cucumber [55,58,59]. Further work is required to identify and understand crops where plateau sensor responses does occur. In these cases, there may be a diminishing sensor sensitivity to increasing crop N status, which could influence the sensitivity of the sensor to identify excessive crop N status [13].

Vegetation indices based on reflectance in the green and in the red edge are reported to be very sensitive to leaf and crop greenness [44,90,150], and have been recommended as being more appropriate for use as crop N indicators than vegetation indices based on reflectance in the red [19,80]. The reason is the higher sensitivity of vegetation indices based on reflectance in the green and red edge to the whole range of chlorophyll content, especially at the high chlorophyll contents that occur with excessive N conditions [95].

## 6. Applications of Proximal Optical Sensors for Crop N Management

Previous sections have dealt with the capacity of proximal optical sensors to evaluate the N status of vegetable crops. The capacity to interpret sensor measurements and apply them to optimal N fertilization is a necessary subsequent step [27]. Considerable research has been done on the development of approaches to use optical sensor measurements for crop N management. Appreciable work has been done with chlorophyll meters; their ease of use, relatively low cost of some sensors, and general sensitivity to crop N status have likely encouraged the development of practical applications. There has also been considerable work on the development of approaches for the use of canopy reflectance sensors for crop N management. Until now, there has been relatively little work developing practical approaches for the crop N management using fluorescence-based flavonols meters.

Absolute (as measured) sensor measurements do not indicate a deficient, sufficient, or excessive crop N status. To interpret sensor measurements in terms of crop N status, two main approaches have been proposed. These approaches are: (i) A relative approach using reference plots or (ii) an absolute approach based on the use of sufficiency values. 

### 6.1. Use of Reference Plots for Crop N Management

A commonly-used procedure is to divide measured values of optical sensors by those measured in a well-fertilized plot without N limitations. The result is the so-called Nitrogen Sufficiency Index (NSI) [151,152]. The basic idea underlying the NSI is that measurements from optical sensors saturate or reach a plateau when there is no N limitation on crop performance; with NSI values < 1 indicating a N deficiency and NSI ≈ 1 indicating a N sufficiency. The NSI approach assumes that luxury N uptake does not occur and that NSI values of >1 are not possible. The use of reference plots has been widely adopted, particularly with cereal crops [57,153,154,155], as a way to reduce the influence of factors, other than N, on the sensor measurement. The effects of various factors, such as abiotic and water stress, disease incidence, and cultivar, on optical measurements will be very similar in both the measured area and the reference plot, thereby, the use of reference plots isolates the effect of the relative N status of the measured area [27,156]. An alternative to the establishment of a reference plot is the virtual reference plot [155], where an area within the field with good growth is assumed not to be N limited (i.e., has sufficient N) and is used for reference measurements.

There are few examples of the use of reference plots in vegetable crops; however, most of the work with reference crops has been conducted in cereal crops [157,158]. Westerveld et al. [57] used chlorophyll meter measurements to aid N fertilization management of cabbage, carrots, and onions. Side-dress N fertilization was applied whenever chlorophyll measurements fell below 95–97% of the values in the reference plot. Regarding proximal canopy reflectance sensors, we are unaware of specific published applications of reference plots with vegetable crops.

Evidence has shown that saturation of optical sensor measurements does not always occur [13] and the adoption of reference plots in these situations may result in the whole farm being considered N deficient if the reference plot has been over fertilized [58].

### 6.2. Use of Absolute Sufficiency Values for Crop N Management

An alternative approach to overcome the limitation of reference plots when saturation does not occur is the use of absolute sufficiency values of optical sensors measurements. Absolute sufficiency values of sensor measurements distinguish between deficiency (below the value), sufficiency (around the value), and excess (above the value) [13], whereas the NSI approach of Section 6.1 does not allow for distinction in the above sufficiency.

Two approaches have, generally, been used to determine absolute sufficiency values: (i) Yield response and (ii) the use of indicators of the crop N status. Generally, yield-based sufficiency values have been derived from linear-plateau segmented regression between optical sensor measurements and yield. Yield-based sufficiency values have been determined, using this approach, for chlorophyll meter measurements in processing tomatoes [53,68] and cucumbers [59], and for canopy reflectance vegetation indices in cucumbers [125]. Absolute sufficiency values based on crop N status have been derived from the relationship between the crop NNI and chlorophyll meter measurements and canopy reflectance vegetation indices for indeterminate tomatoes [55] and cucumbers [59,125,159].

To provide flexibility regarding planting dates, cropping cycles, and location, absolute sufficiency values can be related to the accumulated thermal time [55] and phenological stages [59,77,125]. Sufficiency values for phenological stages have the advantage that they facilitate the use of optical sensors because measurements can be related to easily-recognizable crop development stages [66,77]. The use of chronological age has the limitation that it does not consider differences in crop development caused by different growing conditions of each crop cycle.

The use of absolute sufficiency values may ease crop N management in a straightforward way. When optical measurements deviate from absolute sufficiency values, adjustments should be rapidly made to N fertilizer management to correct for non-optimal crop N status. In a semi-quantitatively approach, this can be done by making adjustments (adding more or less N) to a previous plan of N fertilizer applications [13,53]. The latter is an example of prescriptive-corrective N management [13,160]. In a fully quantitative way, algorithms that implement measured optical sensors values should be developed to calculate recommended N fertilizer corrections following deviation from irrigation and fertigation in vegetable crops [13,22,161], with such a monitoring system enabling the technical capacity of these fertigation systems to spoon-feed N as required by the crop to be fully exploited.

The use of absolute sufficiency values for crop N management with optical sensors may be affected by cultivar and growing conditions. Further research is required to validate sufficiency values with different cultivars and growing conditions [13].

## 7. Selection of Best Proximal Optical Technology for Use on Farms

This review has shown that different optical sensor measurements and indices are generally capable of providing an assessment of crop N status in a wide range of vegetable crops. In scientific terms, the selection of a proximal optical sensor for crop N management should consider various criteria, such as the capacity to provide accurate information of crop N status throughout the entire crop cycle or at critical stages [58]. An important consideration is the crop surface area that is measured. On-the-go measurements with proximal canopy reflectance sensors integrate a larger surface area than leaf clip-on chlorophyll and flavonols meters measurements, thereby, providing a more representative assessment of the crop. Appreciable replication and careful selection of plants to be measured are commonly effective to overcome between-plant heterogeneity inherent with leaf level measurements [21,58].

A very important aspect to take into account when selecting a proximal optical sensor for use by farmers is the cost and ease of use. The high cost of some optical sensors (i.e., above 6000€) makes them unviable for practical use in small vegetable farms and local enterprises, despite their accuracy. Some of these sensors can be considered as being scientific instrumentation that have been developed for research. Recently, different commercial houses have begun to sell more affordable sensors (<3000€) or even low-cost sensors (<1000€) that may represent effective alternatives to research-based sensors for practical use on farms.

Ease of use is another issue to consider when selecting a proximal optical sensor for use on farms. Most sensors provide the value of the different measurements at the same moment of the measurement, but others require the subsequent use of different mathematical equations to calculate the value of the measurement obtained by the sensor. Subsequent data management will make sensors less useful in the practical context as farmers or technical advisors would have to use these equations to obtain the required value.

## 8. Concluding Remarks

This review has highlighted the potential for proximal optical sensors to assist in crop N management of vegetable crops and the practical issues associated with their use. Experience with vegetable crops is more limited than with cereal crops, such as maize and wheat. However, the research conducted with vegetable crops has shown the sensitivity of these sensors to detect crop N status. Chlorophyll meters, canopy reflectance sensors, and fluorescence-based flavonols meters were all able to evaluate crop N status of vegetable crops. The optimal choice of which sensor type for a given cropping situation will depend on the crop, farm characteristics, the grower, the availability of suitable sufficiency values, and technical support. Proximal reflectance sensors measure a large surface area of the canopy, which can appreciably reduce the variability associated with leaf-clip instruments that commonly measure small areas of individual leaves. Most reflectance sensors and some fluorescence-based flavonols meters can make continuous on-the go measurements, thereby, integrating large measurement areas. This may be advantageous in large-scale farms. Compared to passive canopy reflectance sensors, active sensors are insensitive to irradiance conditions, which is an appreciable practical advantage. Chlorophyll and fluorescence-based flavonols meters do not integrate erroneous signals from bare soil (unlike some canopy reflectance sensors), making them more suitable for early crop growth stages and for widely spaced crops.

The use of proximal optical sensors for the evaluation of crop N status has potential for a more sustainable N management of vegetable crops. However, sufficiency values and algorithms that calculate N fertilization recommendations from optical sensors measurements are scarce for vegetable crops. Further research should aim to go beyond merely diagnosing crop N status and suggest precise N fertilizer requirements. It is envisaged that progress will be made in this field in the future, stimulated by the increasing environmental and societal pressure for sustainable crop N management in vegetable crops.

## Figures and Tables

**Table 1:** Characteristics of some proximal optical sensors with potential for use for nitrogen (N) management of vegetable crops.

Sensor Type	Device ^†^	Manufacturer	Measuring Principle	Wavelengths Used (nm)	Scale
Chlorophyll meter	SPAD-502	Konica Minolta (Tokyo, Japan)	Transmittance	650, 940	Leaf
	N-tester	Yara International (Oslo, Norway)	Transmittance	650, 960	Leaf
	atLEAF+	FT Green LLC (Wilmington, DE, USA)	Transmittance	660, 940	Leaf
	MC-100 Chlorophyll Concentration Meter	Apogee Instruments Inc. (Logan, UT, USA)	Transmittance	653, 931	Leaf
	CCM-200 Chlorophyll Content Meter Plus	Opti-Sciences Inc. (Hudson, NH, USA)	Transmittance	653, 931	Leaf
	DUALEX	Force-A (Orsay, France)	Transmittance	710, 850	Leaf
	MULTIPLEX	Force-A (Orsay, France)	Fluorescence	516, 685, 735	Leaf
Reflectance sensor	MSR5/87/16R	CropScan Inc. (Rochester, MN, USA)	Reflectance (passive ^‡^)	460, 510, 560, 610, 660, 710, 760, 810	Canopy
	CropSpec	Topcon Positioning Systems, Inc. (Livermore, CA, USA)	Reflectance (passive)	730-740, 800-810	Canopy
	Spectral Reflectance Sensor	METER Group, Inc. (Pullman, WA, USA)	Reflectance (passive)	532, 570, 650, 810	Canopy
	OptRx Crop Sensor	Ag Leader Technology (Ames, IA, USA)	Reflectance (active ^‡^)	670, 728, 775	Canopy
	N-sensor ALS	Yara International (Oslo, Norway)	Reflectance (active)	670, 730, 760	Canopy
	Crop Circle ACS 430	Holland Scientific (Lincoln, NE, USA)	Reflectance (active)	670, 730, 780	Canopy
	Crop Circle ACS 470	Holland Scientific (Lincoln, NE, USA)	Reflectance (active)	450, 550, 650, 670, 730, 760	Canopy
	RapidScan CS-45	Holland Scientific (Lincoln, NE, USA)	Reflectance (active)	670, 730, 780	Canopy
	GreenSeeker	Trimble Inc. (Sunnyvale, CA, USA)	Reflectance (active)	650, 770	Canopy
	GreenSeeker Handheld	Trimble Inc. (Sunnyvale, CA, USA)	Reflectance (active)	660, 780	Canopy
Flavonols meter	DUALEX	Force-A (Orsay, France)	Fluorescence	375, 650	Leaf
	MULTIPLEX	Force-A (Orsay, France)	Fluorescence	590, 735, 985	Leaf

^†^ Trade or manufacturers’ names mentioned are for information only and do not constitute endorsement, recommendation, or exclusion. ^‡^ Active or passive refers to whether the sensor is fitted or not with an own light source, respectively.

**Table 2:** Most commonly used canopy reflectance vegetation indices for monitoring crop N status. NIR: Near Infrared; FRed: Far red; L: soil brightness correction factor.

Index	Acronym	Equation	Author
Normalized Difference Vegetation Index	NDVI	$\frac{NIR-Red}{NIR+Red}$	Sellers [88]
Green Normalized Difference Vegetation Index	GNDVI	$\frac{NIR-Green}{NIR+Green}$	Ma et al. [90]
Red Ratio of Vegetation Index	RVI	$\frac{NIR}{Red}$	Birth and McVey [91]
Green Ratio of Vegetation Index	GVI	$\frac{NIR}{Green}$	Birth and McVey [91]
Chlorophyll Index	CI	$\frac{NIR}{Red}-1$	Gitelson et al. [92]
Chlorophyll Vegetation Index	CVI	$\frac{NIR}{Green}*\frac{Red}{Green}$	Vincini et al. [93]
Soil Adjusted Vegetation Index	SAVI	$\frac{NIR-Red}{NIR+Red+L}*\left(1+L\right)$	Huete [89]
Optimized Soil Adjusted Vegetation Index	OSAVI	$\frac{NIR-Red}{NIR+Red+0.16}$	Rondeaux et al. [94]
Red Edge Normalized Difference Vegetation Index	RENDVI	$\frac{NIR-Red\;Edge}{NIR+Red\;Edge}$	Gitelson and Merzlyak [95]
Canopy Chlorophyll Content Index	CCCI	$\frac{RENDVI-RENDVI_{min}}{RENDVI_{max}-RENDVI_{min}}$	Barnes et al. [96]
Red Edge Index	REI	$\frac{NIR}{Red\;Edge}$	Vogelmann et al. [97]
Ratio RENDVI/NDVI	RENDVI/NDVI	$\frac{RENDVI}{NDVI}$	Varco et al. [98]
MERIS Terrestrial Chlorophyll Index	MTCI	$\frac{NIR-Red\;Edge}{Red\;Edge-Red}$	Dash and Curran [99]
